# Phenoxodiol sensitizes metastatic colorectal cancer cells to 5-fluorouracil- and oxaliplatin-induced apoptosis through intrinsic pathway

**DOI:** 10.17179/excli2020-2042

**Published:** 2020-06-30

**Authors:** Esra Yaylaci, Hacer Ilke Onen, Atiye Seda Yar Saglam

**Affiliations:** 1 Department of Medical Biology and Genetics, Faculty of Medicine, Gazi University, Besevler, Ankara, Turkey

**Keywords:** apoptosis, colorectal cancer, 5-Fluorouracil, oxaliplatin, phenoxodiol

## Abstract

Colorectal cancer (CRC) is one of the most common types of cancer seen in the world. 5-Fluorouracil (5-Fu) plus Oxaliplatin (1-OHP) remains the backbone of CRC chemotherapeutics, but with limited success. Phenoxodiol (Pxd) is an isoflavone analog with antitumor activity against various types of cancers, and sensitizes chemoresistant cancer cells to chemotherapeutics including platinum and taxanes. This study was, therefore, undertaken to examine whether Pxd pre-treatment with conventional chemotherapeutic agent(s) 5-Fu and 1-OHP co-administration be a therapeutic strategy for CRC. Cell viability and cytotoxicity were evaluated using dimethyl-thiazolyl diphenyl tetrazolium bromide (MTT) and lactate dehydrogenase assays. The percentage of apoptotic and necrotic cells were determined by fluorescence microscopy analysis. Besides, active Caspase-3 levels by ELISA and relative mRNA levels of Caspase 3 (*CASP3*), CASP8 and CASP9 genes were determined by quantitative real-time PCR (qPCR) analysis. The pre-treatment of Pxd followed by 5-Fu and 1-OHP co-administration was more effective at inhibiting cell viability than either chemotherapeutic agents treatment alone. When compared to 5-Fu with 1-OHP alone treatment, Pxd pre-treatment overwhelmingly increased apoptotic Caspase-3 activity levels in CRC cells. Moreover, qPCR analyses showed that *CASP3* and *CASP9* mRNA levels significantly increased after pre-treatment with Pxd followed by 5-Fu and 1-OHP treatments, compared to 5-Fu with 1-OHP alone. Our results suggested that Pxd enhanced the *in vitro* antitumor activity of 5-Fu and 1-OHP. Our study also suggested that Pxd may be a potential candidate agent in advanced CRC and inclusion of Pxd to the conventional chemotherapeutic agent(s) could be an effective therapeutic strategy for CRC.

## Introduction

Colorectal cancer (CRC) is one of the most common types of cancer seen in the world in both sexes and one of the leading causes of cancer-related death in developed countries (Siegel et al., 2019[39]; Miller et al., 2019[30]). The incidence rate of CRC in Turkey (Turkey Cancer Statistics, 2019[42]) is similar to that reported in the United States (Siegel et al., 2019[39]) and it is the third most common malignancy among all cancers.

Chemotherapy, one of the most effective and powerful strategies used in the treatment of CRC, is a cornerstone in cancer treatment for decades. FOLFOX, [5-fluorouracil (5-Fu), folinic acid, oxaliplatin (1-OHP)] or FOLFIRI (5-Fu, folinic acid, irinotecan) is currently standard chemotherapy regimens for the first-line treatment of metastatic CRC (mCRC) (Simpson et al., 2003[41]; Asmis and Saltz, 2008[6]; De Gramont et al., 2000[11]). 5-Fu and 1-OHP remain the backbone of CRC chemotherapeutics, but with limited success that may lead to cancer recurrence. 

The number of potential drug candidates which are capable of re-regulating the sensitivity of CRC cells especially in individuals who relapse or do not respond to treatment, are increasing in recent years. New drug targets are being searched to improve treatment efficacy, and new candidate molecules are being identified (Aguero et al., 2005[2]; Alvero et al., 2007[4]; Gamble et al., 2006[14]; Georgaki et al., 2009[15]; Isono et al., 2018[19]). Therefore, an agent that can sensitize CRC cells to commonly used agents will be useful. So, the use of classical chemotherapy agents, such as 5-Fu and 1-OHP, in combination with newly developed molecules may shed light on new therapeutic approaches for effective CRC treatment. 

Phenoxodiol (Pxd) (also known as idronoxil), is a more potent synthetic analog of plant isoflavone genistein, which inhibits tumor-associated cell surface ubiquinol (NADH) oxidase Type 2 (ENOX2) (Brown et al., 2008[8]). Its inhibition leads to elevation of intracellular NADH levels, and results in yielding of ceramide by sphingomyelinase. Accumulation of ceramide in cell triggers caspase-mediated apoptosis via degradation of X-linked inhibitor of apoptosis protein (XIAP) (Saif et al., 2017[37]).

The effects of Pxd on apoptosis have been studied in different cancer cells such as ovarian (Kamsteeg et al., 2003[21]), head and neck (Aguero et al., 2010[3]), melanoma (Yu et al., 2006[46]), cervical (De Luca et al., 2008[12]), prostate (Aguero et al., 2010[3]; Mahoney at al., 2012[28]), and renal (Isono et al., 2018[19]) cancer. Moreover, Pxd can also trigger cell cycle arrest by upregulating expression of p21 in prostate cancer cell lines (Mahoney at al., 2014[27]). Another important characteristic of Pxd is its chemosensitization properties against chemo-resistant cancer cells. *In vitro* studies have been shown that Pxd sensitizes cancer cells to the antitumor effects of conventional chemotherapeutics in various types of human malignancies (Sapi et al., 2004[38]; Kluger et al., 2007[22]; McPherson et al., 2009[29]; Morré et al., 2009[33]; Yao et al., 2012[45]; Li et al., 2014[25]; Miyamoto et al., 2018[31]). Furthermore, previous studies have also indicated that Pxd can also increase the sensitization of tumor cells to traditional chemotherapeutic agents in xenograft models of various cancer types including ovarian (Alvero et al., 2006[5], 2007[4]), prostate (McPherson et al., 2009[29]), osteosarcoma (Yao et al., 2012[45]), gallbladder (Li et al., 2014[24]) cancer. 

In our current study, we aimed to find out whether, Pxd pre-treatment alone and in combination with 5-Fu and 1-OHP can enhance apoptotic response in both wild type HCT-116^p53+/+^ and mutant HCT-116^p53-/- ^cells. The present study was, therefore, undertaken to examine whether sensitization of Pxd to 5-Fu plus 1-OHP is a therapeutic approach for chemoresistant CRC cells.

## Materials and Methods

### Cell culture conditions and viability assay

The wild type p53 HCT-116 (HCT-116^p53+/+^) human CRC cell line (purchase from ATCC, Rockville, MD, USA) and mutant p53 HCT-116 (HCT-116^p53-/-^) [gift from Dr. Bert Vogelstein (Johns Hopkins, Baltimore, MD)] in DMEM containing 10 % fetal bovine serum (FBS) and supplemented with 1 % L-Glutamine, 1 % antibiotics/antimycotic agents. Cell culture media and other supplies were obtained from GIBCO (Rockville, MD, USA). All cells were maintained at 37 °C in a humidified 5 % CO_2_ incubator and passaged using trypsin/EDTA solution when they reached 80 % confluence.

Pxd, 5-Fu [purchase from Sigma-Aldrich (St. Louis, MO, USA)] and 1-OHP [purchase from Glentham Life Sciences (Edinburgh, UK)] were dissolved in 100 % dimethyl sulfoxide (DMSO) [purchase from Sigma-Aldrich (St. Louis, MO, USA)] to prepare proper stock solutions and stored at -20 °C usage in experiments. The cells were treated with 0.1 % DMSO as a control in all experiments.

Cells were seeded in a 96-well plate containing DMEM supplemented with 1 % FBS at 5x10^3^ cells per well. After overnight culture, cells were incubated with a serial range of 5-Fu (1-400 μM) and 1-OHP (1-100 μM) alone at both 24 and 48 h. According to our data and other studies (De Angelis et al., 2004[9], 2006[10]; Adamsen et al., 2007[1]; Evert et al., 2018[13]; Li et al., 2018[23]; Guo et al., 2006[17]; Lin et al., 2012[25]), 5-Fu (200 µM) and 1-OHP (5 µM) combination (FOLFOX) were selected for 24h due to their intended cytotoxic effect in cells. Cells also were exposed to fixed concentration of Pxd (10 µg/ml) (Kamsteeg et al., 2003[21]; Alvero et al., 2007[4]; Georgaki et al., 2009[15]; Gamble et al., 2006[14]) because of its sensitization effect. After 4 h of Pxd pre-treatment, medium was removed and cells were treated with 5-Fu and 1-OHP for an additional 24 hours. At the end of all incubation periods, 20 μl of a 5 mg/ml stock MTT solution [purchase from Sigma-Aldrich (St. Louis, MO, USA)] was added to each well and incubated 4h at 37°C. The culture medium was then removed and formazan crystals were dissolved in 100 µl (in wells) of DMSO. Then, the absorbance was determined spectrophotometrically at 570 nm using a microplate reader (Spectramax M3; Molecular Devices, CA, USA). All assays were performed 6 replicates in 3 independent experiments.

### Evaluation of cell death by the Acridine orange/Ethidium bromide staining 

The Acridine orange/Ethidium bromide (AO/EtBr) staining method was carried out whether studied agents induce the CRC cells to die by apoptosis or necrosis. This staining method was performed as previously described by Rubins et al. (1998[36]). After staining, cells were observed under a fluorescence microscope (Olympus, Tokyo, Japan) at 40X magnification (excitation wavelength of 590 nm). The CRC cells were counted and classified as viable, apoptotic and necrotic cells. The selected concentration of the agents was reproduced independently at least three times.

### Quantitation of lactate dehydrogenase (LDH) release

Cytotoxicity Detection Kit Plus [purchase from Roche Applied Science (Mannheim, Germany) was used to determine whether the agents have any cytotoxic effect on the CRC cells. To detect the LDH release from necrotic cells into the extracellular fluid, cells were cultured in 96-well plates at 1x10^4 ^cells/well and incubated for 24 h. This assay was performed according to the supplier's instruction. The optical density was measured at 490 nm with a Spectramax M3 microplate reader (Molecular Devices, San José, CA, USA). Concentrations of all agents were performed 6 replicates in 3 independent experiments. The percent cytotoxicity values was calculated according to the formula provided by the manufacturer's protocol.

### Detection of active caspase-3 levels

To detect whether apoptosis is caspase-dependent, cleaved (active) caspase-3 levels were evaluated by PathScan® Cleaved Caspase-3 (Asp175) Sandwich Elisa Kit (Cell Signaling Technology Inc., Danvers, MA, USA) as described by the suppliers. After treatment the selected concentration of the agents on MCF-7 cells, cell lysate was extracted. Protein content was detected by bicinchoninic acid (BCA)™ protein assay kit (Pierce, Rockford, IL, USA). The same protein amount of each sample was applied to cleaved caspase-3 coated wells of the provided plate. At the end of the kit experimental protocol, the absorbance was measured at 450 nm with an microplate spectrophotometer (Spectramax M3). Each test was repeated in duplicate, and mean values were calculated.

### Total RNA extraction and quantitative real-time PCR (qPCR)

As recommendation of supplier, total RNA was isolated from cells after treatment with agents by the use of TRIzol reagent (Invitrogen, UK). The amount of extracted total RNA in each sample was quantified by NanoDrop spectrophotometer (NanoDrop 2000, Thermo Scientific, Massachusetts, USA). The cDNA was synthesized from 1 µg of total RNA using Transcriptor High Fidelity cDNA synthesis kit (Roche Diagnostics GmbH) with random hexamers following manufacturer's instructions. Appropriate gene specific intron spanning caspase 3 (CASP3), caspase 8 (CASP8), caspase 9 (CASP9) and glyceraldehyde-3-phosphate dehydrogenase (GAPDH) [a housekeeping gene] primers were designed by the online Universal Probe Library Assay Design Center (Roche Diagnostics GmbH) (https://lifescience.roche.com/en_tr/brands/universal-probe-library.html#assay-design-center). Then, appropriate probe/primer combination was selected. The selected primer and locked nucleic acids (LNA) probe sequences are depicted in Table 1(Tab. 1). Each qPCR reaction was prepared in triplicate using Light Cycler 480 Probes Master mix (Roche Diagnostics GmbH) and then run on a LightCycler® 480 instrument (Roche Diagnostics GmbH). After detection of the Cp values in each sample, fold changes were calculated using the ΔΔCT method (Pfaffl et al., 2002[34]). 

### Statistical analysis

All data were expressed as the mean values ± standard deviation (SD) and analyzed via Student's t-test. P<0.05 was evaluated as statistically significant difference. Relative Expression Software Tool 2009 v2.0.13 (Qiagen) was used in the analysis of the relative fold change in the levels of mRNA.

## Results

### Effects of Pxd, 5-Fu and 1-OHP on the CRC cell viability

**Figure 1:**
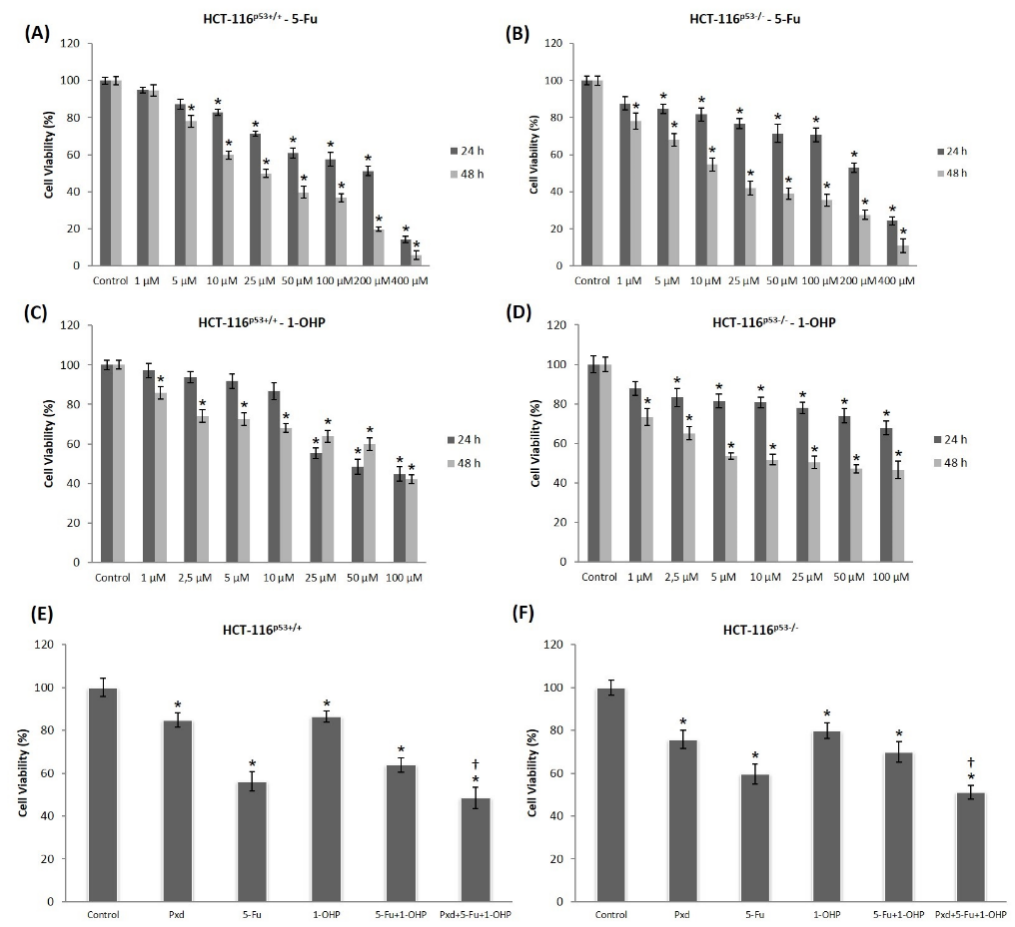
Cell viability of 1-400 μM 5-Fu treated HCT-116^p53+/+^ (A) and HCT-116^p53-/-^ cells (B), 1-400 μM 1-OHP treated HCT-116^p53+/+^ (C) and HCT-116^p53-/-^ cells (D) at 24 and 48 h. The effects of Pxd, 5-Fu and 1-OHP alone treatment, and 10 µg/ml of Pxd for 4 hours; afterward, Pxd was removed from the media and the cells were treated with 200 µM 5-Fu and 5 µM 1-OHP for an additional 24 hours in HCT-116^p53+/+^ (E) and HCT-116^p53-/-^ cells (F). *p < 0.05 compared with control group; ^†^p < 0.05 compared with 200 µM 5-Fu and 5 µM 1-OHP co-treated group; Pxd: Phenoxodiol; 5-Fu: 5-Fluorouracil; 1-OHP: Oxaliplatin

CRC cells were incubated with (1-400 μM) 5-Fu and (1-100 μM) 1-OHP alone at both 24 and 48 h and cell viability of these agents was detected by MTT assay. While HCT-116^p53+/+^ cell viability was decreased at 10 μM or higher concentrations of 5-Fu for 24 h, HCT-116^p53-/-^ cell viability was diminished at 5 μM or greater concentrations. After 48 h of incubation, viability of HCT-116^p53+/+^ and HCT-116^p53-/- ^cells were inhibited at 5 and 1 µM or higher concentrations of 5-Fu, respectively (p<0.05) (Figure 1A-B(Fig. 1)). After 24 h of treatment, we found significantly decreased cell viability in 1-OHP concentrations equal to 25 μM or higher in HCT-116^p53+/+^ cells (p<0.05) (Figure 1C(Fig. 1)). However, in HCT-116^p53-/- ^cells, cell viability statistically decreased in 1-OHP concentrations 2.5 μM or greater (p<0.05) (Figure 1D(Fig. 1)). As shown in Figure 1C-D(Fig. 1), 1-OHP significantly reduced cell viability in both CRC cells at 1 μM and higher concentrations at 48 h (p<0.05). The sensitivity of both CRC cells to 5-Fu and 1-OHP treatments was different except incubation at 48 h with 1-OHP. Compared to untreated control cells, the cells treated with 10 μg/ml Pxd for 24 h, both HCT-116^p53+/+^ and HCT-116^p53-/-^ cells viability decreased by 82 % and 76 %, respectively (Figure 1E-F(Fig. 1)). Furthermore, these cells were also co-administered with selected concentrations of chemotherapeutic agents (200 µM 5-Fu and 5 µM 1-OHP) for 24 h. The decline in viable cells was statistically significant when compared with the untreated control (p < 0.05) (Figure 1E-F(Fig. 1)). Co-administration with 5-Fu and 1-OHP for 24 h overwhelmingly decreased the cell viability to 62 % and 69 %, respectively. To explore whether Pxd could enhance the chemosensitivity of CRC cells to co-administration of 5-Fu and/or 1-OHP, CRC cells were pre-treated Pxd for 4 h; afterward, Pxd was removed from the media and the cells were co-administered with 5-Fu and 1-OHP for an additional 24 h and analyzed cell viability. Our results show that Pxd pre-treatment was more effective in inhibiting cancer cell proliferation than cell death induced by co-administration of 5-Fu and 1-OHP. After Pxd incubation for 4 h, followed by co-administration with 5-Fu and 1-OHP for additional 24 h decreased the viable HCT-116^p53+/+^ and HCT-116^p53-/-^ cells to 51 and 56 %, respectively. Moreover, Pxd pre-treatment resulted in a strikingly diminished cell viability compared to 5-Fu and/or 1-OHP alone treatment in both CRC cells (Figure 1E-F(Fig. 1)).

### Cytotoxic effect of Pxd, 5-Fu and 1-OHP on CRC cells

**Figure 2:**
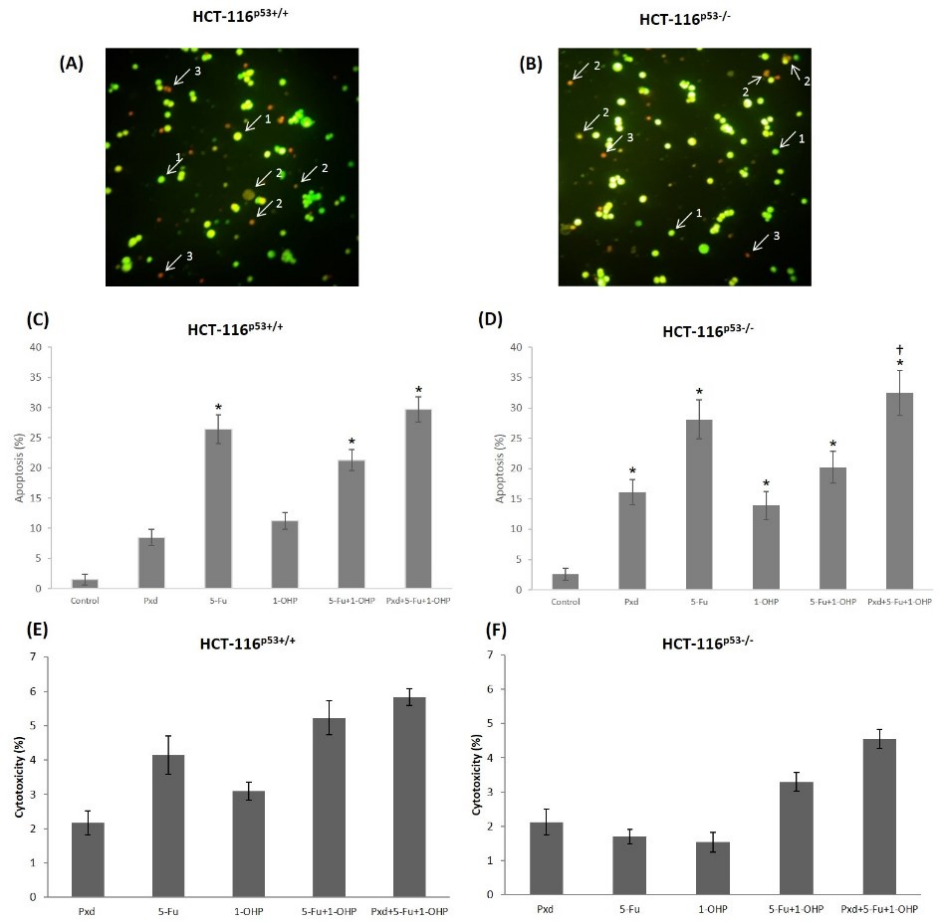
Representative images of viable (1), apoptotic (2), and necrotic (3) cells were determined by AO/EtBr staining using a fluorescence microscope at x400 magnification. HCT-116^p53+/+^ (A) and HCT-116^p53-/-^ cells (B) co-treated with 200 µM 5-Fu and 5 µM 1-OHP at 24 h. Apoptotic ratios of cells treated with Pxd, 5-Fu and 1-OHP alone treatment, and 10 µg/ml of Pxd for 4 hours; afterward, Pxd was removed from the media and the cells were treated with 200 µM 5-Fu and 5 µM 1-OHP for an additional 24 hours in HCT-116^p53+/+^ (C) and HCT-116^p53-/-^ cells (D). Cytotoxic effects of agents on HCT-116^p53+/+^ (E) and HCT-116^p53-/-^ cells (F) determined by LDH release into culture medium. The results are expressed as means ± SD from three independent experiments. *p < 0.05 compared with control group; ^†^p < 0.05 compared with 200 µM 5-Fu and 5 µM 1-OHP co-treated group; Pxd: Phenoxodiol; 5-Fu: 5-Fluorouracil; 1-OHP: Oxaliplatin; AO: acridine orange; EtBr: ethidium bromide

The cytotoxic effects of studied agents on both HCT-116^p53+/+^ and HCT-116^p53-/-^ cells were determined by lactate dehydrogenase (LDH) activity measurement. Cytotoxicity was very low and similar in both CRC cells exposed to selected concentration of Pxd for 24 h (Figure 2E-F(Fig. 2)). As shown in Figure 2E-F(Fig. 2), 5-Fu alone and 1-OHP alone treatments caused an increase in cytotoxicity at 24 h in both CRC cells. Whereas the increase was higher in HCT-116^p53+/+^ cells than the HCT-116^p53-/-^, there was a lack of statistically significant differences (p>0.05; Figure 2E-F(Fig. 2)). Likewise, both co-administration of 5-Fu and/or 1-OHP and Pxd pre-treatment followed by 5-Fu and/or 1-OHP administration was more cytotoxic in HCT-116^p53+/+^ cells than the HCT-116^p53-/-^, although no statistical significance was detected (p>0.05; Figure 2E-F(Fig. 2)). In all experiments, cytotoxicity ratio did not exceed 6 %.

### Detection of morphological cellular changes

In order to detect whether decline in cell viability was because of apoptosis or necrosis, CRC cells were stained with AO/EtBr 24 h after the treatment of cells with the studied agents. AO/EtBr staining indicated that decline in cell viability found in MTT assay resulted from apoptotic response of the cells (Figure 2(Fig. 2)). Representative images of viable, necrotic and apoptotic cells were depicted in Figure 2A-B(Fig. 2). The apoptosis ratio was statistically significant for administrations of 5-Fu alone, 5-Fu and/or 1-OHP co-administration, and Pxd pre-treatment followed by 5-Fu and/or 1-OHP in both CRC cells (p<0.05) (Figure 2C-D(Fig. 2)). Furthermore, Pxd alone and 1-OHP alone treatments were also resulted in statistically significant increases in apoptotic ratio of HCT-116^p53-/-^ cells (p<0.05) (Figure 2C-D(Fig. 2)). In addition, when corresponding to 5-Fu and/or 1-OHP co-administration, Pxd pre-treatment followed by 5-Fu and/or 1-OHP administration elevated apoptotic cells in HCT-116^p53-/- ^(p<0.05) (Figure 2D(Fig. 2)).

### Detection of caspase-dependent apoptosis

**Figure 3:**
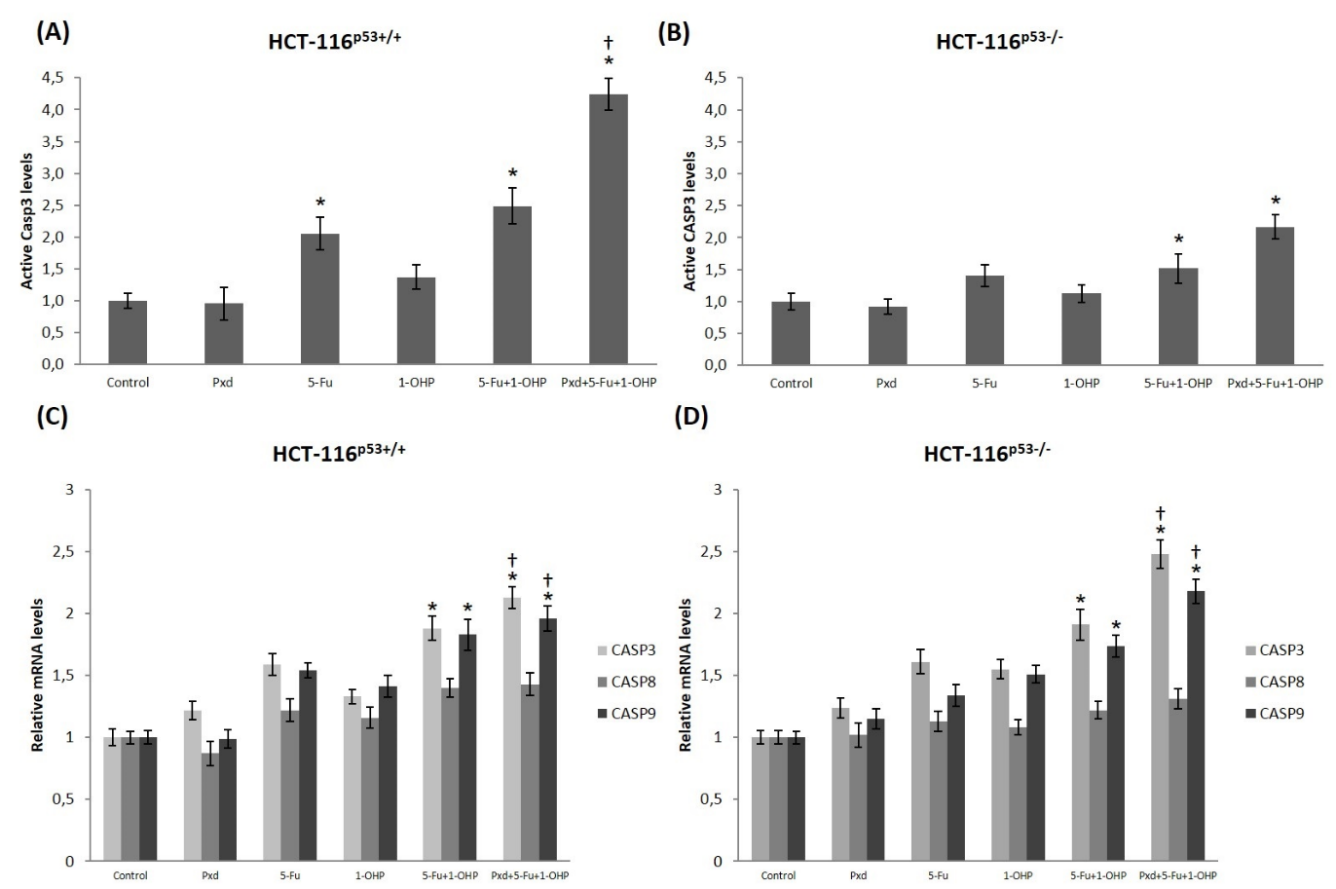
Cleaved caspase-3 protein levels of each agent alone treatment and pretreatment of 10 µg/ml of Pxd for 4 hours; afterward, Pxd was removed from the media and the cells were treated with 200 µM 5-Fu and 5 µM 1-OHP for an additional 24 hours in HCT-116^p53+/+^ (A) and HCT-116^p53-/-^ cells (B). Comparison of the mRNA expression levels of CASP3, CASP8 and CASP9 genes in HCT-116^p53+/+^ (C) and HCT-116^p53-/-^ cells (D) treated with agents at different time points. *p < 0.05 compared with control group; ^†^p < 0.05 compared with 200 µM 5-Fu and 5 µM 1-OHP co-treated group; Pxd: Phenoxodiol; 5-Fu: 5-Fluorouracil; 1-OHP: Oxaliplatin; CASP3: Caspase 3; CASP8: Caspase 8; CASP9: Caspase 9; qRT-PCR: quantitative real-time PCR; mRNA: messenger RNA; PCR: polymerase chain reaction

In order to detect whether apoptosis is caspase-dependent or -independent, we measured the amount of cleaved caspase‐3 in both CRC cells. Only 5-Fu alone treatment in HCT-116^p53+/+^ cells for 24 h, the level of the cleaved caspase-3 protein increased nearly 2 fold and this elevation was statistically significant (p<0.05) (Figure 3A(Fig. 3)). Yet, either single-agent treatment in HCT-116^p53-/- ^cells for 24 h, there was no statistically significant increase in the levels of cleaved caspase-3 protein (p>0.05) (Figure 3B(Fig. 3)). Statistically significant increase in the amount of the cleaved caspase-3 protein was detected for administrations of 5-Fu and/or 1-OHP co-administration, and Pxd pre-treatment followed by 5-Fu and/or 1-OHP in both CRC cells (p<0.05) (Figure 3A-B(Fig. 3)). Moreover, in HCT-116^p53+/+^ cells, Pxd pre-treatment followed by 5-Fu and/or 1-OHP strikingly elevated the level of the cleaved caspase-3 protein when compared with 5-Fu and/or 1-OHP co-administration (p<0.05) (Figure 3A(Fig. 3)). Based on our findings, it can be interpreted that Pxd pre-treatment enhanced caspase‐dependent apoptosis in both CRC cells.

### Effect of Pxd, 5-Fu and 1-OHP treatment on CASP3, CASP8 and CASP9 mRNA levels

In order to detect whether caspase-3 was activated, by either intrinsic or extrinsic apoptotic pathways, the relative mRNA expression levels of *CASP3*, *CASP8* and *CASP9* genes were determined by qPCR. The lack of statistically significant differences in the mRNA levels of *CASP3*, *CASP8* and *CASP9* genes were observed after single-agent treatments for 24 h in both CRC cells (p>0.05; Figure 3C-D(Fig. 3)). Similarly, *CASP8* mRNA expression level was not affected after administration of 5-Fu and/or 1-OHP co-administration, and Pxd pre-treatment followed by 5-FU and/or 1-OHP in both CRC cells (p>0.05) (Figure 3C-D(Fig. 3)). After administration of 5-Fu and/or 1-OHP co-administration in HCT-116^p53+/+ ^cells, the mRNA levels of *CASP3* and *CASP9* were induction by 1.88-fold (P<0.05) and 1.83-fold (P<0.05), respectively. Moreover, in HCT-116^p53-/- ^cells, this treatment resulted in a 1.91-fold (P<0.05) and a 1.74-fold (P<0.05) upregulation of *CASP3* and *CASP9 *mRNA levels, respectively. When Pxd pre-treatment was followed by 5-Fu and/or 1-OHP administered to the HCT-116^p53+/+ ^cells, the expression levels of the *CASP3* and *CASP9 *increased 2.48-fold (P<0.05) and 2.18-fold (P<0.05), respectively. Similarly, in HCT-116^p53-/- ^cells, this treatment resulted in elevation of the *CASP3* [2.13-fold, (P<0.05)] and *CASP9 *[1.96-fold, (P<0.05)] mRNA expression levels, when compared with the control cells. Furthermore, when corresponding to 5-Fu and/or 1-OHP co-administration, Pxd pre-treatment followed by 5-Fu and/or 1-OHP administration to both CRC cells increased the mRNA expression levels of *CASP3* and *CASP9* and, these elevations were statistically important (P<0.05).

## Discussion

The fate of CRC treatment depends on the use of targeted therapy that could disrupt survival pathways and activate cell death pathways. The resistance to anticancer drugs that arise due to the increase of selective tumor cell groups during the treatment, in tumor tissues which initially responds to standard chemotherapy, is one of the major causes of cancer progress. For this reason, the number of potential drug candidates which are capable of re-regulating the sensitivity of CRC cells especially in individuals who relapse or do not respond to treatment, are increasing in recent years. 5-Fu and 1-OHP are frequently used in combined regimens for CRC (Adamsen et al., 2007[1]; Asmis and Saltz, 2008[6]; De Angelis et al., 2006[10]; De Gramont et al., 2000[11]; Ikehata et al., 2014[18]; Simpson et al., 2003[41]). In particular, chemotherapeutic agent combinations that are currently used are not effective in patients with unresectable and metastatic CRC (Van Cutsem et al., 2016[43]). Therefore, studies investigating newly developed chemical agents to be used alone or in combination with classical chemotherapy agents, such as 5-Fu and 1-OHP, are needed for the effective treatment of CRC. Additionally, the resistance to chemotherapeutic agents and the cytotoxic effects of high concentrations of these agents draw attention to the investigation of different molecules (Berindan-Neagoe et al., 2013[7]). 

Epidemiological studies that showed an inverse relationship between isoflavone consumption and cancer risk have drawn attention to the value of these components in cancer treatment (Mahoney et al., 2012[28]; Silasi et al., 2009[40]). Pxd, a novel synthetic isoflavone derivative, has been shown to have a particularly potent effect on reversing chemotherapeutic resistance. Pxd inhibits proliferation in a wide range of human cancer cells such as leukemia, breast, ovarian and prostate and it is 20 times more potent than genistein (Jin and El-Deiry, 2005[20]). Additionally, Pxd has an impact on survival and death pathways and administered in combination with standard chemotherapeutics or monotherapy in solid cancers. In our study, we aimed to investigate if Pxd pre-treatment alone and in combination with 5-Fu and 1-OHP can effect apoptotic, cytotoxic and antiproliferative response in both wild type HCT-116^p53+/+^ and mutant HCT-116^p53-/- ^cells. 

In our study, it was observed that 5-Fu and 1-OHP decreased the viability in both HCT-116 cvell lines at 24h. Based on cell viability results, 200 μM 5-Fu and 5 μM 1-OHP concentrations in 5-Fu and 1-OHP combinations where similar effects were investigated in various cancer cell lines were determined as reference (De Angelis et al., 2004[9], 2006[10]; Adamsen et al., 2007[1]; Evert et al., 2018[13]; Li et al., 2018[23]; Guo et al., 2006[17]; Lin et al., 2012[25]). For this purpose, it was shown that the cell viability rate for 200 μM 5-Fu was approximately 56 % and 60 %, and for 5 μM 1-OHP was 87 % and 80 % in HCT-116^p53+/+^ and HCT-116^p53-/-^ cells, respectively. For Pxd, the viability in both CRC cells was found to be approximately 80 % with the recommended concentration and time (10 μg/mL, 4 hours) in the literature (Kamsteeg et al., 2003[21]; Alvero et al., 2006[5]; Gamble et al., 2006[14]). After pre-treatment of 10 μg/ml Pxd to the cells for 4 hours, Pxd was removed from the medium and 200 μM 5-Fu with 5 μM 1-OHP was co-administered for 24 hours. When viability rates of 5-Fu and 1-OHP alone or in combination with Pxd pre-treated cells were compared, viability rates of pre-treated cells were significantly reduced. The viability of both CRC cells decreased significantly after treatment with 5-Fu and 1-OHP followed by Pxd treatments, compared to 5-Fu with 1-OHP alone. Accordingly, in both CRC cells, after a pre-treatment of 10 μg/ml Pxd for 4 hours, the cell viability rate was approximately 49 % and 51 % after removal of Pxd from the medium and co-administration of 200 μM 5-Fu and 5 μM 1-OHP for 24 hours in HCT-116^p53+/+^ and HCT-116^p53-/-^ cells, respectively. Based on these data, we can say that Pxd pre-treatment makes CRC cells more sensitive to the combination of 5-Fu and 1-OHP. No studies have been found in the literature regarding the co-administration of 5-Fu and 1-OHP following Pxd pre-treatment to HCT-116^p53+/+^ and HCT-116^p53-/-^ cells, so we could not compare our findings. On the other hand, consistent with our results, in the studies using various cancer cell lines, it has been reported that Pxd makes these cells more susceptible to different agents and standard chemotherapeutic drugs (Kamsteeg et al., 2003[21]; Alvero et al., 2006[5]; Gamble et al., 2006[14]; Georgaki et al., 2009[15]; Kluger et al., 2007[22]; Silasi et al., 2009[40]; Aguero et al., 2005[2]). Also, several studies showed that Pxd rearranges sensitivity by significantly lowering IC_50_ values against paclitaxel, carboplatin, gemcitabine, docetaxel and topotecan in ovarian epithelial cancer cells (Kamsteeg et al., 2003[21]; Alvero et al., 2006[5]; Silasi et al., 2009[40]). In addition, when Pxd was removed from the environment after 2 hours of pre-treatment of 10 μg/ml in ovarian cancer cells, it increased sensitivity against chemotherapeutic agents such as carboplatin, paclitaxel and gemcitabine that were administered for 24 h, by decreasing cell viability rates and increasing apoptotic effect (Alvero et al., 2006[5]). Moreover, this proapoptotic effect has been shown to occur only in cancer cells and does not cause any toxicity in normal ovarian epithelial cells (Kamsteeg et al., 2003[21]; Alvero et al., 2006[5]). In another study investigating the effects of Pxd on human umbilical vein endothelial cells (HUVEC), it was shown that Pxd inhibits tumor cell growth and proliferation as well as antiangiogenic effect (Gamble et al., 2006[14]). When the effects of Pxd on different cancer cells was investigated, it was found that Pxd stimulates cancer cells to apoptosis, terminal differentiation and mitotic arrest (G1 phase). Based on these findings, Pxd was granted Fast Track status by the Food and Drug Administration (FDA) as a re-regulator of chemotherapeutic susceptibility for platinum and taxanes in the treatment of recurrent ovarian and hormone-resistant or non-resistant prostate cancers (Gamble et al., 2006[14]; Wang et al., 2012[44]). These results indicate that the pre-treatment of Pxd in combination with other chemotherapeutic agents may be more beneficial in achieving the most effective concentration and time and in sensitizing chemotherapeutics with known effects against CRC cells, rather than by administering it alone in cancer cells.

The CRC cells were stained with AO/EtBr to reveal and evaluated morphologically whether the molecular mechanism underlying the decrease in cell viability stemmed from apoptosis or necrosis. Human CRC cells were stained with AO/EtBr and evaluated morphologically with the fluorescence microscope. Accordingly, it was determined that Pxd pre-treatment in both CRC cell lines directed cells to apoptosis more than necrosis. When the cell ratios leading to apoptosis were compared, HCT-116^p53-/-^ cells (apoptotic ratio 32.3 %) were found to be more sensitive than HCT-116^p53+/+^ cells (apoptotic ratio 29.7 %). It was observed that the cell viability rates were consistent with our fluorescence staining results. These findings were also confirmed by LDH release into the medium. Our results are in line with other studies that showed the cytotoxic effects of Pxd in both HCT-116 cells. Results of various studies performed with Pxd application to different cancer cell lines showed that cytotoxic effect after Pxd application did not change significantly, similar to our study (Mahoney et al., 2012[28]; Alvero et al., 2006[5]; Georgaki et al., 2009[15]).

Targeting the factors regulating apoptosis is a current approach to the treatment of cancer cells (Reed, 2002[35]) and fundamental feature of a cancer cell is its resistance to undergo apoptosis (Lowe and Lin, 2000[26]; Mor et al., 2002[32]), thus, reversing this resistance to apoptosis shows the best approach for treatment of cancer. Apoptosis has been classified into two main groups: an intrinsic and extrinsic pathway (Green and Evan, 2002[16]). The proteolytic active caspase-3 enzyme involved in apoptosis belongs to the group of effector caspases and causes the division of numerous cytoplasmic and nuclear components to reveal the morphological and biochemical properties of the intrinsic pathway of apoptosis. The enzyme caspase-3 as a marker of apoptosis confirms that the cell has undergone apoptosis at the protein level (Kamsteeg et al., 2003[21]; Kluger et al., 2007[22]; Alvero et al., 2006[5]; Gamble et al., 2006[14]; Ikehata et al., 2014[18]). In our study, 5-Fu and/or 1-OHP co-administration, and Pxd pre-treatment followed by 5-Fu and/or 1-OHP in both CRC cells were found to have more active caspase 3 enzymes than untreated control cells. When compared to 5-Fu with 1-OHP alone treatment, Pxd pre-treatment overwhelmingly increased apoptotic activity in CRC cells. Increased protein-caspase-3 activity in both extrinsic and intrinsic apoptotic pathways suggests that both cascade pathways lead to proteolytic degradation using the caspase pathway. Our results are consistent with various studies investigating apoptotic responses in different cancer cells treated with Pxd and show cell-specific apoptotic response when looking at the effects of Pxd on different cancer cell lines (Kamsteeg et al., 2003[21]; Kluger et al., 2007[22]; Alvero et al., 2006[5]). For example, in a study performed with ovarian cancer cell lines, it was observed that the administration of carboplatin, paclitaxel and docetaxel alone for 24 hours did not cause any difference in active caspase-3 activity. After 2 hours of 10 μg/mL Pxd pre-treatment, Pxd was removed and paclitaxel, carboplatin and docetaxel were administered for 24 hours, it was shown that p17 and p19 protein levels, which are the markers of active caspase-3 forms, and caspase-3 activity were increased (Alvero et al., 2006[5]). In another *in vitro* study, carboplatin alone in melanoma cells did not show any effect on XIAP and other components of the apoptotic cascade, but after pre-treatment of 10 μg/mL Pxd for 2 hours and removal of Pxd was followed by carboplatin administration for 24 hours, Caspase 2 and Bid activation and XIAP degradation were observed and a significant increase in caspase-3 activity was reported (Kluger et al., 2007[22]). These results indicate that pre-administration of Pxd alone or in combination with other chemotherapeutic agents results in a similar effect by increasing the level of active caspase-3 in different cancer cells, thereby activating caspase-dependent apoptosis, leading to the death of cancer cells. The presence of active caspase-3 in both CRC cells as a result of Pxd pre-treatment is indicative of induction of caspase-mediated apoptosis in these cells. 

The expression levels of CASP3, CASP8 and CASP9 genes were also investigated by qPCR method to determine in both HCT-116 cell lines. We also demonstrated that CASP3 and CASP9 mRNA levels increased after treatment with 5-Fu and 1-OHP followed by Pxd treatments, compared to 5-Fu with 1-OHP alone. Our results show that Pxd is able to fully activate CASP3, CASP8 and CASP9 in the same cells in which 5-Fu and 1-OHP failed. This suggests that the apoptotic pathway is functional in CRC cells but is activated only in response to Pxd. Although there is no study in the literature regarding the effect of Pxd on mRNA expression levels of CASP3, CASP8 and CASP9 genes HCT-116^p53+/+^ and HCT-116^p53-/-^ cells, active caspase-8, caspase-9 and caspase-3 activities were increased by inducing Fas-dependent apoptotic pathway of 24 hours of Pxd administration in various ovarian cancer cells have been shown to increase (Kamsteeg et al., 2003[21]). In addition, another *in vitro* study, showing the change in apoptotic markers observed at different time intervals after treatment of CP70 ovarian cancer cell line with 10 μg/mL Pxd, showed the degradation of proapoptotic Bak protein at 16 hours post-treatment, significant increase in caspase-8, caspase-9 and caspase-3 activity and the presence of p30 XIAP (Alvero et al., 2006[5]). These data show that Pxd and 5-Fu with 1-OHP induce caspase-dependent apoptosis by showing a synergistic effect.

To the best of our knowledge, our study is the first study to evaluate the ability to suppress proliferation and stimulate apoptosis by the co-administration of 5-Fu with 1-OHP chemotherapeutic agents following Pxd pre-treatment in both HCT-116 human CRC cells. Our results indicate that 5-Fu and 1-OHP render both CRC cells more sensitive to Pxd effects. Therefore, this study suggests that the addition of Pxd to 5-Fu and 1-OHP could potentially be a therapeutic strategy for CRC. If our findings are supported by further molecular analyses in other preclinical *in vitro* and *in vivo* cancer models, we think that Pxd may be a good candidate molecule for classical chemotherapeutic agents in the treatment of CRC.

## Acknowledgement

This study was supported by the Gazi University Projects of Scientific Investigation, with the project code number 01/2018-09. 

## Conflict of interest

The authors declare that they have no conflict of interest.

## Figures and Tables

**Table 1:**
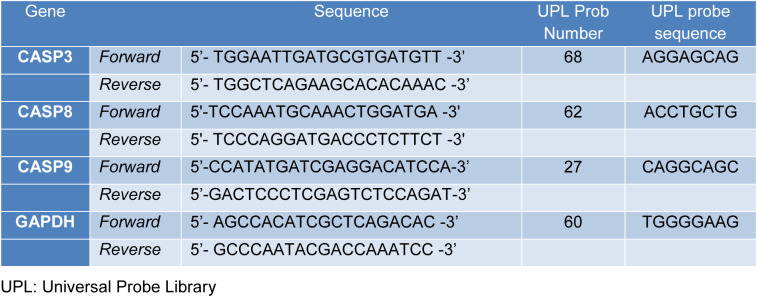
Gene-specific primer sequences, UPL probe numbers and sequences
